# Electrostatic polarization fields trigger glioblastoma stem cell differentiation†

**DOI:** 10.1039/d2nh00453d

**Published:** 2022-11-17

**Authors:** Tamara Fernandez Cabada, Massimo Ruben, Amira El Merhie, Remo Proietti Zaccaria, Alessandro Alabastri, Enrica Maria Petrini, Andrea Barberis, Marco Salerno, Marco Crepaldi, Alexander Davis, Luca Ceseracciu, Tiziano Catelani, Athanassia Athanassiou, Teresa Pellegrino, Roberto Cingolani, Evie L. Papadopoulou

**Affiliations:** Istituto Italiano di Tecnologia via Morego 30 16163 Genova Italy remo.proietti@iit.it enrica.petrini@iit.it teresa.pellegrino@iit.it paraskevi.papadopoulou@iit.it; Department of Electrical and Computer Engineering, Rice University 6100 Main Street Houston TX 77005 USA; Istituto Italiano di Tecnologia via Melen 83 16152 Genova Italy

## Abstract

Over the last few years it has been understood that the interface between living cells and the underlying materials can be a powerful tool to manipulate cell functions. In this study, we explore the hypothesis that the electrical cell/material interface can regulate the differentiation of cancer stem-like cells (CSCs). Electrospun polymer fibres, either polyamide 66 or poly(lactic acid), with embedded graphene nanoplatelets (GnPs), have been fabricated as CSC scaffolds, providing both the 3D microenvironment and a suitable electrical environment favorable for CSCs adhesion, growth and differentiation. We have investigated the impact of these scaffolds on the morphological, immunostaining and electrophysiological properties of CSCs extracted from human glioblastoma multiform (GBM) tumor cell line. Our data provide evidence in favor of the ability of GnP-incorporating scaffolds to promote CSC differentiation to the glial phenotype. Numerical simulations support the hypothesis that the electrical interface promotes the hyperpolarization of the cell membrane potential, thus triggering the CSC differentiation. We propose that the electrical cell/material interface can regulate endogenous bioelectrical cues, through the membrane potential manipulation, resulting in the differentiation of CSCs. Material-induced differentiation of stem cells and particularly of CSCs, can open new horizons in tissue engineering and new approaches to cancer treatment, especially GBM.

## Abstract 2

Glioblastoma cancer stem-like cells seeded on substrates exhibiting surface potential differences, undergo differentiation due to the forced hyperpolarization of the membrane potential at the cell/substrate interface.

## Graphical abstract

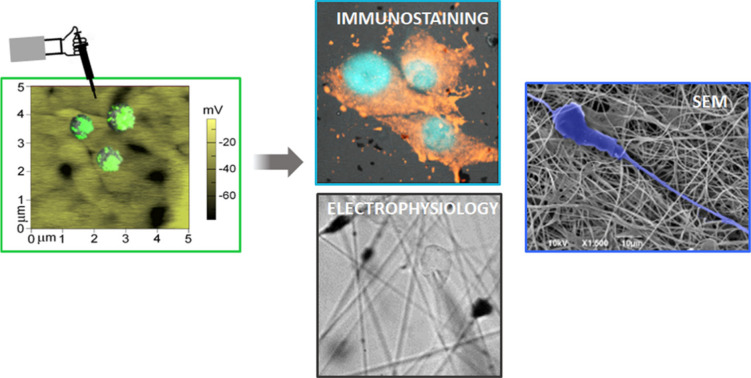

**New concepts**

We show that we can tailor the membrane potential of quiescent cancer stem-like cells (CSCs) of brain tumor, by providing a suitable electrical milieu, *i.e.* an electrospun insulating polymer fibres substrate with embedded electrically conductive graphene nanoplatelets, resulting in surface polarization fields. While it is known that during stem cell differentiation the cell membrane hyperpolarizes, in this work, we show for the first time that the electrostatic fields generated by the substrate lead to the hyperpolarization of the cell membrane and trigger differentiation. We use fundamental physical approaches to provide a theoretical understanding of stem cell differentiation due to electrostatic fields at the cell/material interface, and we prove experimentally that the bioelectrical properties of stem cells can be tailored by fundamental interactions at the electrical interface between cells and materials. Numerical simulations show the change of the *V*_mem_ with the applied fields, which is supported experimentally by electrophysiology. All experimental results show that differentiation of the CSCs took place after a few days *in vitro* only to the polymeric composite substrates with electrostatic fields present (as opposed to pure polymeric ones). The importance of our findings lies in that we elucidate the role of *V*_mem_ in stem cell differentiation.

## Introduction

Every living cell is separated from its exterior by a bilayer membrane, within which various ion pumps and channels are present, balancing the ionic concentrations between the exterior and interior parts of the membrane. The electrical charge imbalance between the inner and outer parts of the cell membrane gives rise to a potential difference, called the membrane potential, *V*_mem_. Membrane potential influences many cell functions, such as proliferation, migration, differentiation and apoptosis.^1–6^

Generally, *V*_mem_ values of differentiated cells lie between −10 mV and −90 mV,^7^ while undifferentiated stem cells, including cancer stem-like cells (CSCs), have more depolarized *V*_mem_ values.^8^ A cell is considered to be depolarized when *V*_mem_ is less negative (*i.e.* closer to zero), and hyperpolarized when *V*_mem_ is more negative than its usual values. It has been shown that hyperpolarization is necessary for stem cell differentiation, while depolarization favours the maintenance of quiescent state of CSCs^8^ and cell proliferation of other cell types.^3,8,9^ It has been shown that pharmacological hyperpolarization of *V*_mem_ triggers differentiation in human myoblasts^10,11^ and in human cardiomyocyte progenitor cells.^4^ Furthermore, pharmacological control of *V*_mem_ can modulate differentiation of human mesenchymal stem cells,^2^ documenting that endogenous bioelectric signals represent driving forces for important cell functions. It is also noteworthy that electrical charges have been shown to modulate the *V*_mem_ in neurons^12,13^ and bacteria.^14^ In addition, scaffolds of graphene-based polymer composites have been shown to allow neurons to retain their physiological *V*_mem_, in contrast to pure polymer ones.^15^

Electric field stimulation has long been known to enhance growth, differentiation and proliferation of stem cells (SC),^16,17^ with external wiring normally used to stimulate the cell culture. Advances in materials science and nanotechnology have enabled the design of materials that can deliver specific cues, such as mechanical, chemical or other stimuli, to the cell and influence its fate.^18^ In this realm, electrically conductive materials have been interfaced with SCs, resulting in a boost in their differentiation.^19–24^ However, in all those studies, growth factors have been used to initiate the SC differentiation (according to SC differentiation protocols), restricting the action of electrical stimulation to auxiliary.

Graphene is a material that has been extensively used to enhance SC differentiation to both neurons^25–27^ and osteoblasts,^28^ depending on the differentiation protocol used. This has been attributed to the physicochemical characteristics of graphene that enhance SC adhesion. Electrostatic interactions due to C–F bonds have also been credited for better adhesion and differentiation of SC onto the graphene substrate.^29^ Other distinctive properties of graphene, such as high electrical conductivity and flexibility, make it suitable for development of multifunctional biomedical devices,^30^ and thus, a possibly potent material for tissue engineering.^31^

Summarizing the above considerations, one is guided to think that (i) *V*_mem_ can be a driving force that can possibly trigger SC differentiation, (ii) external electrical charges can modulate *V*_mem_ and (iii) the cell/material interface may be designed in a way to deliver the specific electrical cues to the cells. Hence, we ask:

Can we use localized polarization fields within scaffolds to induce polarization phenomena in the cell membrane of adherent SCs to trigger differentiation without the use of chemical/biological means?

To this end, we have used polymer matrices with embedded graphene nanoplatelets (GnP) to create electrical polarization fields in the scaffolds. As a model stem cell culture, we have chosen human glioblastoma cancer stem-like cells (CSCs), isolated from human glioblastoma tumor spheres (U87 cell line).

Glioblastoma (GBM; World Health Organization grade IV glioma) is the most prevalent and lethal primary brain tumor.^32^ Unlike other solid tumor cell types, GBM widely invades the surrounding brain but rarely metastasizes to other organs. In recent years, much effort has been invested to fight GBM, especially using targeted therapies, immunotherapies, or more advanced nanotechnology-based therapies, such as magnetic hyperthermia.^33–36^ GBM therapy remains focused on achieving maximal surgical resection followed by concurrent radiation therapy combined with intravenous and oral chemotherapy. Tumor heterogeneity of GBM, containing among others self-renewing, tumorigenic CSCs, has been related to tumor initiation and therapeutic resistance. CSCs function within a local microenvironment, both by actively remodeling the surroundings and by exchanging critical maintenance signals from their niches.^37^ CSCs are defined by their functional characteristics, such as asymmetric division, self-renewal, and tumor initiation ability. CSCs and somatic SCs share some key properties, such as self-renewal and quiescence.^38^ Targeting CSCs to induce changes from highly malignant quiescent CSCs into differentiated cells with low tumorigenicity, may facilitate the effective therapeutic treatment of GBM cells. Here, it is of utmost importance that normal brain cells and neural SCs are not affected during the process. Few studies have reported the interaction of graphene-based materials with the GBM, in 3D cell cultures. It has been shown that graphene oxide promotes CSC differentiation,^39–41^ it provides a dose dependent cytotoxicity,^42^ and it inhibits adhesion, proliferation and migration of CSCs.^43–45^ In addition, specificity of graphene-based materials has been shown.^39^ We should note here that in the aforementioned cases, mixed, CSC-enriched, cell populations were used and at least in one case the authors claimed that in ultra-low attachment seeding conditions, non-CSCs undergo anoikis, a special kind of apoptosis cells in suspension can go through.^39^ In these cases, the CSC differentiation has been attributed to the modulation of various signaling molecules involved in cell survival, cell proliferation, cell death and differentiation by induction of low level of oxidative stress or interference with molecular signaling pathways on the cell surface that communicate with the cell nucleus.

Herein, we have fabricated PA66 (polyamide 66) and PLA (poly(lactic acid)) fibre scaffolds using electrospinning, either pure or with embedded GnP to create polarization fields in the scaffolds. First, we have performed physico-chemical characterization of the samples, including Kelvin probe for the measurement of the polarization fields on the scaffold surface. Embedding GnP in the PA66 (or PLA) matrix, leads to the formation of a polar solid surface with localized charge distributions. We have performed numerical simulations that show how the *V*_mem_ can be tailored by an underlying electric field, due to accumulation of electrical charges. We have used Fluorescent-Activated Cell Sorting (FACS) to isolated CSCs GBM from tumor spheres (containing mixed populations of CSCs and non-CSCs), and we have cultured the pure CSC populations on the various scaffolds, monitoring their morphology by SEM at different days in culture. Finally, at the time points at which morphological evidence suggested differentiation, we have performed electrophysiology measurements to determine the *V*_mem_ of the cells, while immunostaining was used to study Nestin (CSC marker), S100b (early glial marker) and glial fibrillar acidic protein (GFAP; mature glial marker) expression.^46^

The results suggest that CSCs cultured on PA66/GnP and PLA/GnP fibre mats adhere and differentiate spontaneously to the glial phenotype after *ca.* 7–10 days in culture (days *in vitro*, DIV), although they were cultured in quiescent conditions, by adding in the cell culture medium epidermal growth factor (EGF), fibroblast growth factor (FGF) and the cocktail of growth factors contained into B27 supplement (CSC maintenance) – rather than differentiation conditions. In contrast, CSCs could barely adhere on the pure polymer scaffolds, and differentiation was scarcely – if ever – observed. The differentiation process was confirmed by cell morphological studies, electrophysiological measurements and immunostaining confocal analysis. Based on our results, we propose that the spatially heterogeneous electrostatic fields present on the scaffold surface affect locally the membrane potential and are able to trigger differentiation.

## Results and discussion

**Fig. 1:**
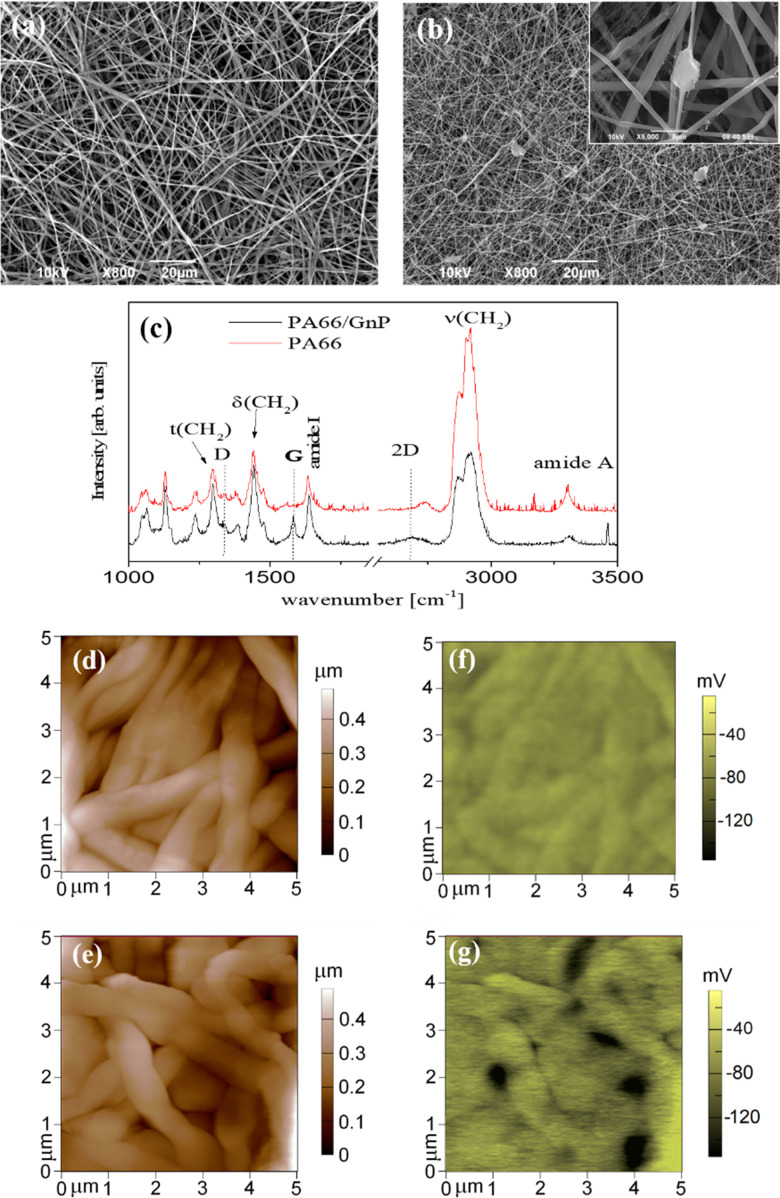
SEM image of (a) PA66 and (b) PA66/GnP fibres. In the inset of (b) a SEM image of high magnification depicts a representative image of GnPs embedded in the polymer matrix. In (c) the μRaman spectra of PA66 and PA66/GnP fibres is shown. AFM topography images of the fibres are shown in (d) for PA66 and (e) for PA66/GnP, with their corresponding electrical surface potential maps, as measured by Kelvin-probe, in (f) and (g). The vertical colour bars on the right show the range of mapped for each image.

PA66 is a material commonly used in life science labs,^47^ easy to fabricate, with favorable mechanical properties.^48^ The fibrous meshes of PA66/GnP are shown in Fig. 1a and b. The average diameter of the PA66 fibres is ∼800 nm, while it reduces down to ∼240 nm for PA66/GnP, due to the increased conductivity of the PA66/GnP solution during electrospinning^49^ (Fig. S1, ESI†). Micro-Raman spectroscopic analysis of the pure and the composite scaffolds (Fig. 1c) shows the fingerprint peaks of PA66 (1636 cm^−1^ for the amide I, 1296 cm^−1^ for the CH_2_ twisting mode, 1445 cm^−1^ and 2908 cm^−1^ the bending and stretching modes respectively of CH_2_, and 3300 cm^−1^ the amide A) and GnPs (1345 cm^−1^ for the D peak, 1585 cm^−1^ for the G peak and 2691 cm^−1^ for the 2D peak), as described in ref. 48. Furthermore, TGA analysis confirms the existence of 8 wt% GnPs in the scaffold (Fig. S2a, ESI†). The GnPs are either incorporated within the fibres or, due to their larger lateral diameter, protrude from the fibres, as shown by SEM (Fig. 1b) and TEM imaging (Fig. S2c and d, ESI†). Such protrusions have been suggested to potentially pierce cell membranes, but without causing substantial membrane disorder.^50^ Mechanical testing shows similar mechanical properties for both scaffolds (Fig. S3, ESI†). Finally, AFM measurements (Fig. 1d and e) show uniform fibres comprising the mats.

The key difference here is that the GnP incorporation in the PA66 matrix results in a marked heterogeneity of surface electrostatic potential of the PA66/GnP scaffold, described in Fig. 1e and g. The surface potential distribution of the PA66 scaffold, as measured by the Kelvin probe mode of an AFM, appears uniform and close to zero, whereas the surface potential map of the PA66/GnP scaffold comprises numerous light and dark regions, corresponding to the insulating polymer and the conductive GnP fillers, respectively, and reaching values up to 150 mV. Thus, incorporation of GnPs creates local variations in charge distribution on the scaffold.

In order to evaluate the effects of local charge distribution of PA66/GnP on a CSC membrane, some 3D numerical simulation have been conducted. In particular, we focused on investigating the conditions needed to drive the membrane potential from values close to zero to more negative values, a process that describes hyperpolarization of the cell membrane. We have chosen as starting point a *V*_mem_ of −10 mV (for the undifferentiated CSCs), and as endpoint (*i.e.* the differentiation value) a *V*_mem_ of −50 mV (indicative values^9^).

**Fig. 2:**
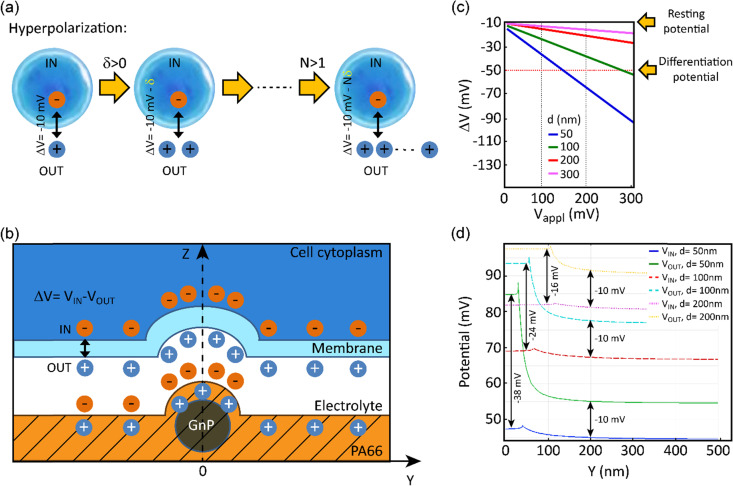
(a) Hyperpolarization explained graphically: the cell cytoplasm (dark blue) is negatively charged. If we define Δ*V* = *V*_IN_ − *V*_OUT_ as the potential difference between the two sides of the membrane (the membrane is indicated by a lighter blue line surrounding the cell), Δ*V* becomes more negative by increasing the number (*N*) of positive charges (*δ*) on the external side of the membrane, as shown in the diagram. (b) Unit cell representing the PA66/GnP scaffold (not in scale). The surface of the composite is positively charged (as measured by our digital electroscope), with higher density of charges in the spots where GnPs are present (as measured by Kelvin probe). The positive charges outside the cell membrane are a consequence of the formation of a capacitor when the cell is placed on the positively charged surface. Zero charge boundary conditions are applied. (c) The calculated membrane potential for GnP electric potential, *V*_appl_, ranging from 0 mV to 300 mV. (d) The membrane potential is evaluated separately for both the internal (IN) and external (OUT) membrane boundaries, along the *Y* axis, *i.e.* further away from the GnP, for a constant *V*_appl_ of 100 mV. In both (c and d) it is assumed *e*_cytoplasm_ = 80 with GnP diameter *d* equal to 50, 100, 200 and 300 nm (the latter case for (c) only).

This electric potential change, Δ*V*, describing a hyperpolarization process, can be achieved by adding positive electrical charges on the external side of the cell membrane (Fig. 2a). In our modeling, this condition is realized by imposing specific electrical potential values to both sides of the membrane. More specifically, we have modeled our PA66/GnP scaffold as a spherical core of GnP surrounded by PA66. Furthermore, on top of PA66, an electrolyte layer of 20 nm thickness is found,^51^ followed by a 10 nm thick cell membrane separating the electrolyte layer from the cell cytoplasm. This description is depicted in Fig. 2b and it matches well with the capacitor model introduced in Schoen and Fromherz's work,^51^ as the elements membrane, electrolyte and PA66/GnP can indeed be considered forming a capacitor. In particular, in the composite regions where GnPs are present a decreased surface potential, with respect to the GnP-free regions, is measured by the Kelvin probe.

The sign of charges on the surface of the PA66/GnP substrate (Fig. 2b) was measured to be positive, as expected by the triboelectric table,^52^ by a built-in-house digital electroscope (Fig. S4, ESI†). The optical permittivities used for the different materials employed in the calculations are: *ε*_GnP_ = 10, *ε*_membrane_ = 2.5, *ε*_cytoplasm_ = 80 [ref. 53] and 200 [ref. 54], *ε*_PA66_ = 3.6 while the electrolyte is assumed as an ideal ion conductor (a hypothesis that does not affect the general conclusions of our modeling). The GnP core is represented by a sphere of variable diameter (*d* = 50, 100, 200 and 300 nm) in order to take into account the GnP sub-domains as previously reported in ref. 55. During the simulation, we assume as a starting point that the GnP core is uncharged while the resting membrane potential of the adjacent CSC is −10 mV. Under this assumption, an external potential applied to the GnP core can induce accumulation of charges directly in the GnP core, resulting in surface potential differences, as described experimentally in Fig. 1g. Once this is performed, a change in the membrane potential is achieved. More specifically, Fig. 2c depicts the variation of the membrane potential, Δ*V*, for the four different GnP core diameters *versus* externally applied positive potential (*i.e.*, corresponding to an increase of positive charges) on the GnP core. Our numerical simulations suggest that the smaller the GnP diameter, the stronger the hyperpolarization effect, hence making it easier to achieve the differentiation condition (here assumed to be −50 mV).

Finally, Fig. 2d shows the potential calculated along the internal and external membrane boundaries, as a function of distance of the membrane from the GnP core (*Y* axis), when 100 mV are applied to GnP. As shown in Fig. 2b, close to the GnP core Δ*V* attains more hyperpolarised values (more negative values), while far away from the GnP core, where the GnP charges are not felt by the membrane, Δ*V* equals −10 mV (the starting *V*_mem_ value, representing the *V*_mem_ of CSCs). For the 50 nm diameter GnP, Δ*V* reaches a value of −38 mV close to the GnP core, while for bigger GnP diameters Δ*V* attains smaller values. Furthermore, when *ε*_cytoplasm_ is increased from 80 to 200, the achieved potential is even closer to the −50 mV membrane potential goal (data not shown). These results suggest that the inclusion of charged GnPs in the polymer matrix can induce localized electric fields on the surface of the scaffold, resulting in local electrical changes of the cell membrane, finally leading to the hyperpolarization of the cell membrane.

**Scheme 1:**
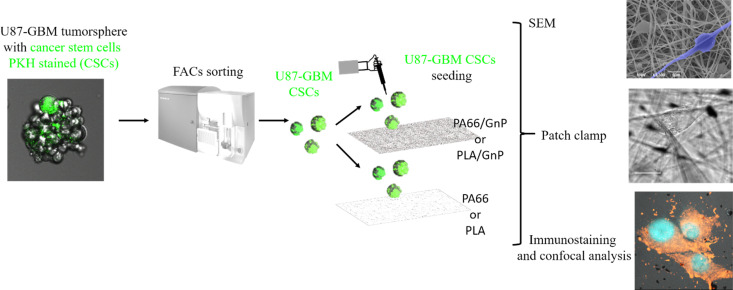
U87 GBM tumorspheres were stained with PKH lipophilic dye. After DIV7–10 of cell proliferation, only quiescent cells (CSC) keep the green PKH staining. PKH stained cells were then sorted by FACS and they were seeded on the scaffolds for SEM analysis, immunostaining with confocal analysis and electrophysiology.

In order to sort the CSC sub-population prior to seeding it on the PA66/GnP substrates, we used a PKH diluting dye assay to stain and then sort by Fluorescent-Activated Cell Sorting (FACS) the quiescent (and thus stained) CSCs from mixed cell populations of U87 GBM tumor spheres, as described in Scheme 1 and the Methods.^56,57^

**Fig. 3:**
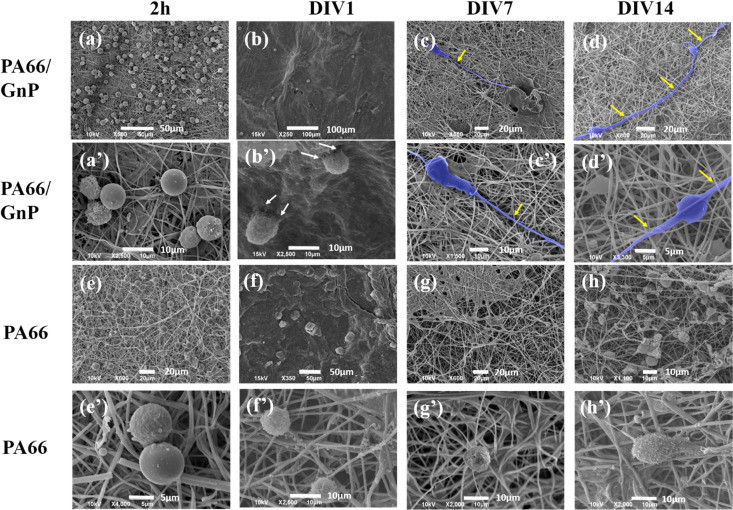
SEM images of CSCs cultured on PA66/GnP in lower and higher magnification, for (a and a′) 2 h, (b and b′) DIV1, (c and c′) DIV7 and (d and d′) DIV14. In 2 h and DIV1, cells have rounded morphology, while at DIV7 and 14, cells are elongated with long axons, assuming typical glial morphology (false blue colour). Similarly, for CSCs on pure PA66 fibres, for (e and e′) 2 h, (f and f′) DIV1, (g and g′) DIV7 and (h and h′) DIV14, cells maintain their undifferentiated rounded shape at DIV14, and no signs of differentiation is evident from the SEM images. Note: we did not always find cells in the pure PA66 fibre mats, and generally, CSC adherence on those samples was much less compared to the PA66/GnP, probably washed away during sample preparation and this is why in (g) no cells are present.

In Fig. 3, representative SEM images depict CSC cultures on the composite (PA66/GnP) and pure polymeric (PA66) fibre mats at different time points, specifically for 2 h and DIV 1, 7 and 14. After 2 h cells exhibit round morphology on both PA66/GnP and PA66 substrates, while at DIV1 cells seeded on PA66/GnP mats have developed small protrusions to adhere to the substrate (shown by white arrows). At DIV7 and DIV14, cells on the PA66/GnP mats have attained the typical morphology of adhering cells, and cell transformation with clear spreading cell bodies, less roundish and more adhering on the substrate and the presence of filament protrusions, evidencing signs of cell differentiation. Indeed, cells have developed long, thick, filamentous protrusions typical for the glial phenotype (indicated by yellow arrows in Fig. 3). Control CSC cultures for the same time points on pure PA66 fibre mats do not show evidence of strong attachment on the substrate: at DIV1 cells have a rough surface full of small vesicles, a typical sign of cell suffering while at DIV14 cells maintain their round morphology, and do not show morphological signs of differentiation.

In order to proceed with electrophysiology and confocal microscopy experiments, the transparency of the sample is crucial, because these techniques rely on light transmittance through the sample. Hence, we have fabricated new electrospun samples, based on GnPs and a transparent polymer – poly(lactic acid) (PLA) and thin layers of fibres were electrospun on coverslips. PLA was chosen as a typical biomaterial,^58,59^ also presenting positive charges, according to the triboelectric tables^60^ and as confirmed by built-in-house digital electroscope (data not shown). Fibre diameters are comparable to the PA66 and PA66/GnP ones (Fig. S5a and b, ESI†). AFM and Kelvin probe measurements (Fig. S5c and d, ESI†) showed the presence of a surface potential difference of approximately 200 mV, due to the presence of GnPs, comparable to the data of PA66/GnP samples.

**Fig. 4:**
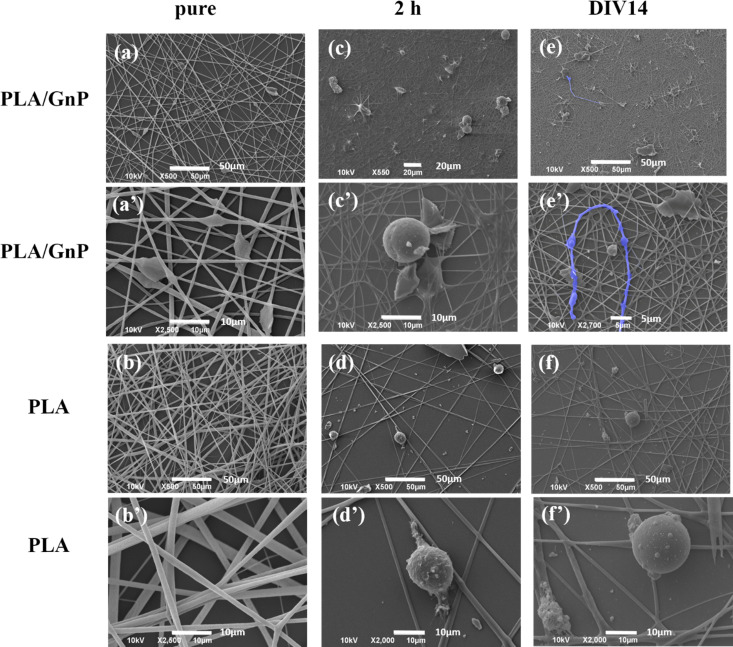
SEM images of PLA and PLA/GnP fibre mats, either (a and a′) and (b and b′) pristine or with CSC cultures for (c and c′) and (d and d′) 2h and (e and e′) and (f and f′) 14 days. CSC grown on pure PLA maintain their rounded morphology even after 14 days in culture, while GSC grown on the PLA/GnP composite mats present an elongated morphology with long protrusions (false blue colour), morphological evidence of differentiation to glial phenotype.

SEM imaging of CSC cultures on PLA/GnP and PLA electrospun fibres on coverslips, for either 2 h or DIV14 (Fig. 4), documented significant morphological evidence of differentiation to the glial phenotype, when CSCs were seeded on the PLA/GnP substrates, while the cells maintained the rounded morphology of the CSCs on the bare PLA substrates, even on DIV14. These SEM results confirmed the results obtained on PA66.

**Fig. 5:**
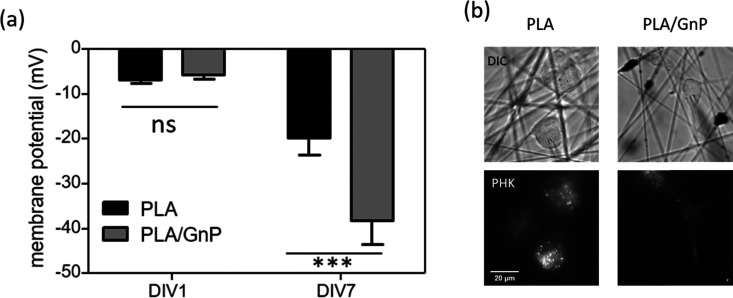
(a) Membrane potential of single U87 CSCs seeded on PLA or PLA/GnP substrates at DIV1 and DIV7. *N* = 19–24 cells per experimental condition. One way ANOVA followed by Bonferroni post test; ***p < 0.001). (b) Representative DIC images of cancer stem cells grown on PLA and PLA/GnP and their corresponding PKH staining. Scale bar: 20 μm. The patch pipette used to measure the cell's membrane potential is visible in the DIC images.

According to our simulation, the polarization fields present on the fibres due to the GnP presence on the polymer matrix should lead to *V*_mem_ hyperpolarization. Electrophysiology measurements were performed on CSC cultures using the patch clamp technique at DIV1 and DIV7. CSCs grown on both PLA and PLA/GnP substrates showed a markedly depolarized membrane potential at DIV1, *i.e.* less than −10 mV, as expected for CSCs,^8,61,62^ evidencing their stemness (Fig. 5). Interestingly, at DIV7 only cells on PLA/GnP fibres showed a distinctly hyperpolarized membrane potential, of 38.1 ± 5.3 mV. On the other hand, the membrane potential of CSCs seeded on pure PLA fibres was mildly hyperpolarized to 19.7 ± 3.9 mV. This mild hyperpolarization of the cells on PLA fibres, might be due to charge retention in the PLA fibre mats, after the electrospinning process, resulting in a slightly positively charged substrate.^63^

These results confirm that the presence of GnPs in the polymer matrix affects the progression of the resting membrane potential of the CSCs. Since CSCs were cultured in quiescent conditions that do not normally allow differentiation (presence in the cell culture medium of EGF, FGF and B27 supplement growth factors), we can ascribe the hyperpolarization of the membrane potential of the CSCs to the spatially inhomogeneous electric fields on the surface of the fibres, as described by our model.

Furthermore, a quantification study of the cell morphology (elongated *vs.* rounded cells) was carried out on bright field images taken on the same samples destined to immunostaining and confocal analysis (see for example Fig. 5b, lower, right panel). The results, presented in Fig. S7 (ESI†), clearly show a statistically significant difference between the percentage of elongated cells with respect to the total amount of cells on the PLA/GnP fibres and the percentage of elongated cells found on PLA fibres, highlighting the effect of the electrically conductive fillers in the substrates. It is interesting to note the progressive increase in the percentage of elongated cells attached to PLA/GnP fibres with time, decreasing only at DIV10. This decrease is probably due to long culturing time for those cells which exceeded the optimal culturing time (which is 4 days in the experimental conditions^64^). Meanwhile, a small increase in the percentage of elongated cells is also observed on PLA fibres, following the same trend as the cells on PLA/GnP, *i.e.* this number decreases at DIV7.

**Fig. 6:**
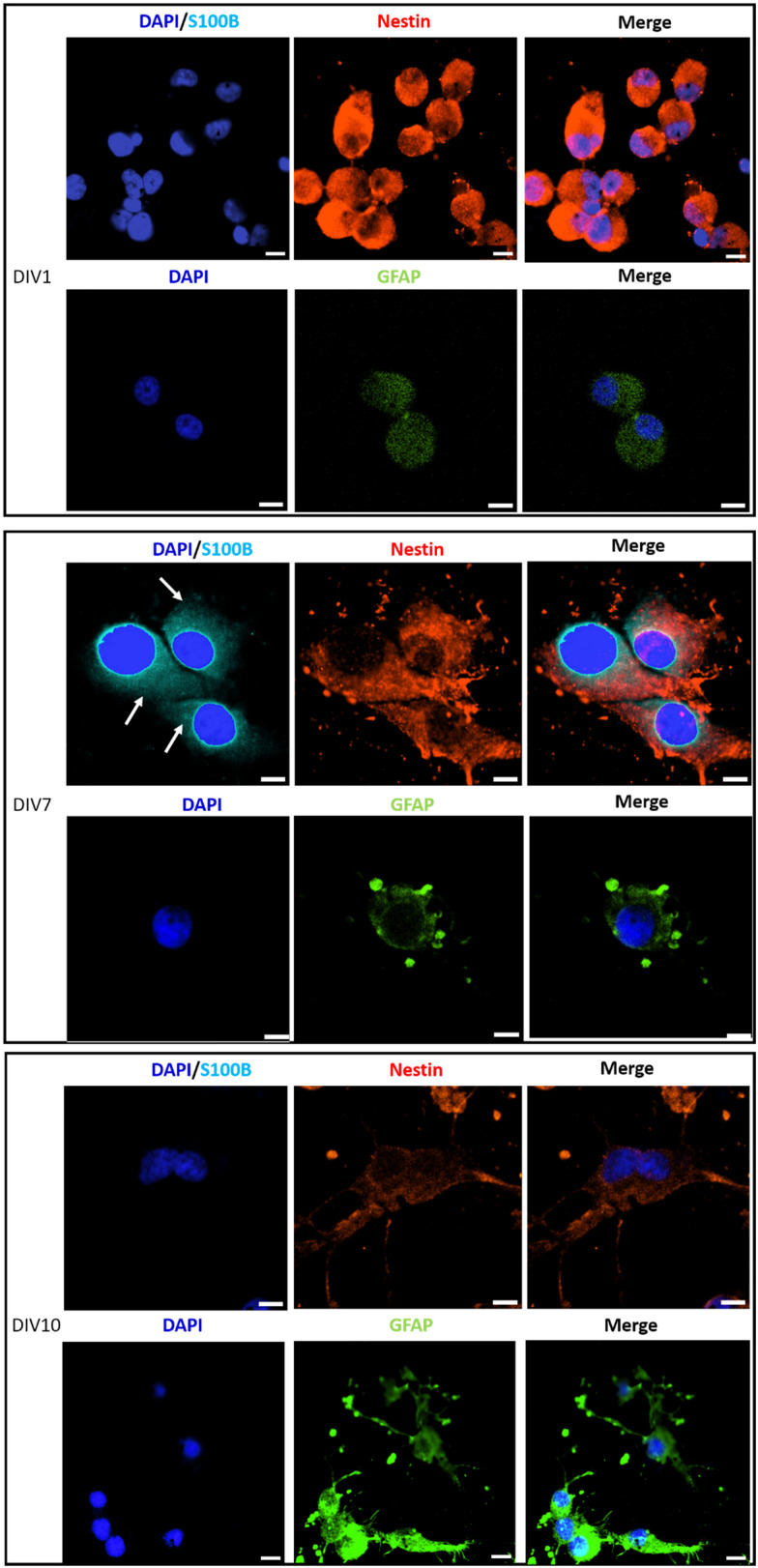
Immunostaining of sorted U87 CSCs, seeded on PLA/GnP substrates, analyzed by immunofluorescence and confocal microscopy, on DIV1, DIV7 and DIV10. Cell nuclei are stained by DAPI (blue, false colour). S100B protein is stained in light blue color. Nestin intermediate filament protein is stained in red color. GFAP marker is shown in green (false color). Antibody anti-S100B is used to label glial cells phenotype at the beginning of cell differentiation process (immature status; expressed into the cell cytoplasm on DIV7, light blue color, white arrows). Antibody anti-Nestin is used to stain cells coming from CSCs (red). Antibody anti-GFAP (green false colour) is used to stain glial-phenotype cells in a mature status of cell differentiation. Scale bar: 10 μm. Note: we could not find cells adhered on pure PLA substrates. This is probably due to the harsh treatment that the samples had to go through during the preparation for confocal microscopy.

Immunostaining analysis of the expression of stemness and differentiation-phase markers at different time points of the culture, has contributed to a deeper understanding of the effect of PLA/GnP substrates on CSCs differentiation (Fig. 6). Nestin was used as a marker for stemness as previously reported,^65^ S100B as an early glia differentiation marker,^46^ and GFAP as a mature glia marker.^46^ At DIV1, cells seeded onto PLA/GnP show a high expression of Nestin, as expected for CSCs (Fig. 5). In addition, the absence of fluorescence signal from glial markers (S100B and GFAP) indicates the absence of glial cells at this time point. We should also note that at DIV1, all cells maintain their rounded shape. However, at DIV7, a S100B marked expression is observed in the cytoplasm, accompanied by a moderate expression of Nestin and a light increasing expression of GFAP was detected on elongated cells, indicating loss of stemness and an early stage of differentiation towards a glial phenotype. Furthermore, at DIV10 and for cells seeded onto PLA/GnP substrates, the Nestin expression decreases notably, indicating the loss of cell stemness. In addition, the absence of S100B expression into the cytoplasm, with a concomitant increase in the GFAP expression strongly indicates differentiation to the glial phenotype.^46^ Morphologically, at DIV10 the cells have attained an elongated shape, similar to the morphology seen by SEM (Fig. 3 and 4), compatible with the glial phenotype. Finally, we should note that in none of the immunostaining experiments performed were we able to find cells on the pure PLA fibre mats. This is probably due to poor adhesion of the CSCs on the pure PLA, combined with the mechanical stress due to the multiples washing steps of the sample preparation protocol for confocal analysis.

## Conclusions

The cumulative results obtained by SEM, electrophysiology and immunostaining, highlight the pivotal effect of the GnPs embedded in polymer matrices, on the differentiation of GBM CSCs towards the glial phenotype. We have argued that the presence of GnP-associated surface charges dynamically change the cell membrane potential, triggering differentiation. Numerical simulations show the tendency of the *V*_mem_ to hyperpolarize with increasing potential of the substrate underneath the CSCs, while the direct quantification of *V*_mem_ at ∼−40 mV is a clear documentation of the hyperpolarization of the CSCs membrane potential.

The surface polarization fields present on the cell/material interface is a fundamentally physical event that can be considered as first messenger. This implies that following events are required to transduce this message downstream and activate signaling pathways leading to differentiation. The current knowledge of this signaling cascade needs further investigation. Nevertheless, differentiation of CSCs is considered as a crucial therapeutic target, to facilitate the drug treatment of resistant CSCs upon their differentiation. This work is a proof-of-concept on how the electrical interface between cells and materials can be a powerful tool to tailor the bioelectric properties cancer stem-like cells, driving their differentiation.

## Methods

### Materials

Polyamide 66 (PA66) was obtained from Sigma-Aldrich (molecular weight 120 000, degree of polymerization of 531, density 1.14 g mL^−1^). Poly(lactic acid) (PLA) was obtained from NatureWorks (PLA 6060D). Graphene nanoplatelets (herein GnP) were kindly provided by Directa Plus PLC (grade Ultra G+). According to the company specifications, GnPs have lateral dimensions of few tens of micrometres and a thickness of tens of nanometres. Reagent grade solvents, trifluoroacetic acid (TFA) and acetone were purchased from Sigma Aldrich and used without further purification. PKH dye was purchased from Sigma Aldrich. Dulbecco's modified Eagle's medium: nutrient mixture F-12 (Gibco™, UK), B-27 supplement (1×) (Gibco™, UK), basic fibroblast growth factor (PeproTech), epidermal growth factor (PeproTech), Accutase enzyme (Sigma-Aldrich), bovine serum albumin (Sigma), Triton X-100 (Sigma), PBS 1× buffer (Sigma), anti-glial fibrillary acidic protein rabbit antibody (Sigma), anti-nestin mouse antibody (Invitrogen), anti-S100B rabbit antibody (Sigma), anti-rabbit antibody Texas red conjugated (Invitrogen), anti-mouse antibody Texas red conjugated (Invitrogen), anti-rabbit antibody Alexa Fluor 405 conjugated (Invitrogen), DAPI (Roche).

### Electrospinning

Solutions for electrospinning were prepared by diluting PA66 pellets in TFA : acetone solution, at 1 : 1 volume ratio,^48^ to obtain a concentration of 15 wt% polymer in solution, at room temperature. In the case of PLA, the polymer was in a CHCl_3_ : DMF solution at volume ratio of 8 : 2 and concentration of 11 wt% polymer in solution. In the case of the composite fibres, 10 wt% GnP were added to the respective solutions and probe sonicated (750 W, 40% amplitude, 20 kHz, using a Sonics and Materials, Inc, Model Num VCX750). A plastic syringe with a stainless steel 23-gauge needle (18-gauge needle in the case of PLA) was filled with the solution and connected to a syringe pump (NE-1000, New Era Pump Systems, Inc.), working at a flow rate of 200 μl h^−1^ (400 μl h^−1^ in the case of PLA). The solution was electrospun at a voltage of 14 kV (18 kV in the case of PLA), controlled by a high voltage power supply (EH40R2.5, Glassman High Voltage, Inc.). The PA66 and PA66/GnP fibres were collected on an aluminum plate placed at a distance of 16 cm from the needle. The PLA and PLA/GnP fibres were collected on circular coverslip glasses of diameter 18 mm (for immunostaining experiments) or 13 mm (for electrophysiology experiments).

### Morphological characterization

The morphology of the produced fibres was characterized by scanning electron microscopy (SEM, JEOL JSM-6490LA, Japan, Tokyo), working in high vacuum mode, with an acceleration voltage of 10 kV, after they had been coated by a thin gold layer (15 nm). PA66/GnP fibres were also characterized by transmission electron microscopy (TEM) in order to understand how the GnPs are accommodated in the PA66 fibres. In order to analyze the samples with TEM a specific preparation was required. Fibrous samples were infiltrated for 8 hours and then embedded in low viscosity Spurr (SPI-Chem) epoxy resin. Once the resin has hardened (48 h in the oven at 65 °C), 150 nm thick sections were cut with a Leica EMU C6 ultra-microtome and placed onto Cu grids. TEM images were collected with a Jeol JEM 1011 (JEOL, Japan, Tokyo) electron microscope, operating at an acceleration voltage of 100 kV, and recorded with a 11 Mp fibre optical charge-coupled device (CCD) camera (Gatan Orius SC-1000).

### Chemical characterization

The crystal structure was studied by X-ray diffraction (XRD) using a Rigaku SmartLab X-ray diffractometer, equipped with a 9 kW Cu Kα (*λ* = 1.542 Å) rotating anode, operating at 40 kV and 150 mA. A Göbel mirror was used to convert the divergent X-ray beam into a parallel beam and to suppress the Cu Kβ radiation (*λ* = 1.392 Å). The diffraction patterns were collected at room temperature, over an angular range of 4° to 35°, with a step size of 0.05° and scan speed of 1.2° min^−1^. μRaman spectra were collected at ambient conditions using a Horiba Jobin Yvon LabRAM HR800 μRaman spectrometer, equipped with a microscope. A 632.8 nm excitation line, in backscattering geometry through a 50× objective lens, was used to excite the specimens, at low power of 0.25 mW. The experimental set-up consists of a grating 600 lines per mm with spectral resolution of approximately 1 cm^−1^. Thermal studies were carried out using thermogravimetric analysis (TGA). The degradation temperature of the materials was evaluated by TGA. During TGA measurements, samples were heated from 30 °C to 600 °C at a heating rate of 10 °C min^−1^ under nitrogen atmosphere set at a flow rate of 50 mL min^−1^.

### Atomic force microscopy and scanning Kelvin probe microscopy

Scanning Kelvin probe microscopy (SKPM) is a variant of atomic force microscopy (AFM). Simultaneously with the main AFM scan being carried out in normal tapping mode to track and map the surface topography, after each scan line – interlaced with it – the tip is moving at constant distance Δ*H* over the previously mapped surface (lift pass), and the electric surface potential is detected. This is done according to the Kelvin probe principle. In this setup, the tip and the sample surface are the plates of a capacitor and the tip is oscillating up and down with a given (small) electrically-driven amplitude above the sample. The force on the tip due to the electric field between the plates is canceled out by a feedback loop, which adds to the tip (the sample is set to ground) an equal DC voltage like the surface potential. An MFP 3D (Asylum Research, CA, US) AFM was used for these measurements, with a MESP probe (Bruker, MA, US), having nominal resonance frequency and tip apex diameter of 75 kHz and 70 nm, respectively, the latter being due to a ∼25 nm thick CoCr coating. The maps were acquired at 40 × 40 μm^2^ scan area (256 × 256 pixels) with 0.5 Hz line frequency; the elevation height for the lift pass was Δ*H* = 100 nm. Due to the fibrous nature of the sample, and in order to avoid trapping of the cantilever in the fibrous mat and its consequent crash during the scan, the samples had to be pressed with 3.5 ton for 3 min (sample diameter 1 cm) in order to obtain a relatively flat top sample surface. This resulted in a slightly deformed image of the surface, where the fibres have mostly lost their round geometry and/or their diameter is slightly bigger than the one measured by SEM.

### Mechanical properties

The mechanical properties of electrospun mats were characterized through uniaxial tensile tests on a universal testing machine (Instron 3365) equipped with a 10 N load cell. Handling of the samples was facilitated by the paper frame method. Briefly, samples were carefully cut (15 × 2 mm^2^) and mounted on a disassemblable paper frame, from there transferred to the machine clamps, where the paper frame was opened to release the strip samples. All tests were performed with the loading rate of 0.1 mm min^−1^ (strain rate 0.1 min^−1^) at laboratory conditions (21 ± 1 °C, 50 ± 5 RH%). Three repetitions were conducted for each material. From the resulting stress strain curves, the apparent Young's modulus *E*, ultimate tensile strength and elongation were extracted.

### Digital electroscope

We implemented a solid-state prototype of an electrostatic detector to determine the sign of the charges on the samples surface. The detector comprises a single depletion Metal Oxide Semiconductor Field Effect Transistor (MOSFET) LND150N3, and an industrial Light Emitting Diode (LED) L-793SRD-D, connected in series. MOSFET detectors are normally used in literature to implement Electric Field Imaging (EFI) systems.^66^ The field effect transistor gate capacitance *C* is charged through a variation of the voltage *V* of a sensing electrode with respect the circuit local ground, based on the fundamental law *I* = *C*d*V*/d*t*, where *I* is the current flowing though the capacitor. Once a positive or negative charged object approaches the gate terminal of the transistor, current *I* is generated, therefore charging or discharging the gate capacitance, *C*. Based on the resulting gate-source voltage, the transistor modulates the current flowing through the LED, I_LED, thus the emitted light based on the approached charge. For positive charges the light increases, whereas for negative charges the light decreases. Note that the circuit operates under dynamical conditions, because (i) current is a function of the derivative of the voltage, (ii) the transistor, that internally provides an Electro Static Discharge (ESD) sub-circuit, has a leakage that discharges the gate capacitance, (iii) the AC power distribution field perturbates the stored gate capacitance charge. The DC bias point of the transistor is manually reset using a non-latching switch. Notwithstanding the gate capacitance of the circuit is systematically perturbed by the AC power distribution source (50 Hz frequency), in any case this effect does not visually impact on the generated average light.

### Cell culture

U87 glioblastoma multiforme (U87 MG) cell line (kindly provided by Dr Emilio Ciusani from Istituto Neurologico Carlo Besta, Milano) was cultured in 3D model in Corning® ultra-low attachment U-flasks (CORNING, USA) in a 5% CO_2_ humidified incubator at 37 °C. Cells were grown in Dulbecco's modified Eagle's medium: nutrient mixture F-12 (DMEM/F12) (Gibco™, UK) freshly supplemented with B-27 supplement (1×), 20 ng mL^−1^ basic fibroblast growth factor, and 10 ng mL^−1^ epidermal growth factor. Half of the cell culture medium was changed to a fresh one every 3 days. The cells with these conditions are enriched with cancer stem cells (CSC) and fast proliferating tumoral cells (non-CSCs), forming U87 tumorspheres. U87 tumorspheres were splitted every 3 days following the dissociation procedure described in the following.

### Cell membrane labeling: PKH staining

In order to sort the CSCs from the non-CSCs, we performed cell membrane labeling by the lipophilic green fluorescent dye PKH67 (Sigma-Aldrich) (Fig. S6, ESI†). The labeling was performed on the cells passage number (60–69) that had been in culture for at least 1 week after defrosting them. The cells were stained with PKH67 dye following the provider's protocol. Briefly, tumor spheroids were collected and pelleted by centrifugation, followed by their incubation with Accutase (Sigma-Aldrich) at 37 °C for 15 min for dissociation. Subsequently, the cells were pelleted again and incubated with the dye ethanolic solution for 4 min (1 : 500 dye *versus* dilution buffer volume). The staining reaction was quenched by adding fetal bovine serum (FBS). Finally, the cells were washed once with DMEM/F12-1% FBS (without growth factors) and finally diluted in DMEM/F12 supplemented cell culture medium and cultured on Corning® ultra-low attachment U-flasks. Half of the medium was changed every 3 days for a total of 10 days cultures and the cells examined daily by an optical microscope (Motic AE31, Country) to confirm the spheroid formation (Fig. S8, ESI†). To follow the fluorescent signal dilution, images were captured using a Nikon inverted microscope TiE equipped with a confocal microscope (Nikon Optical Co., Ltd, Japan) at excitation wavelengths *λ* = 488 nm.

### Fluorescent-activated cell sorting (FACS) of PKH-stained cells

Following PKH67 staining, the cells were subcultured for 7–10 days. FACS (FACSAriaII, BD Biosciences) was used in order to isolate the PKH^pos^ cell population. We isolated the most epifluorescent cells accounting for the 0.2–0.4% of the total cell population (PKH^pos^ cells, gated at 103–104 fluorescence units) and based on size. Furthermore, we found out that PKH^pos^ cells (U87 CSCs) based on forward scattering (FSC), which is an indirect measurement of size, usually form a distinct cell population with large size (≥20 or 30 μm) in comparison with PKH^neg^ cells (<10 μm) size. Typically PKH^pos^ cells represent <0.1% of the total mixed cell population. Following sorting (generally between 100 000 and 200 000 cells), cells were diluted in 5 ml of DMEM/F12 cell culture medium on ice.

### Cell cultures on scaffolds

Following FACS, the PKH^pos^ cells (U87 CSC) were seeded on the PA66/GnP and pure PA66 scaffolds or on PLA/GnP and PLA coated 10 mm diameter glass coverslips. In the case of PA66/GnP and PA66 substrates, the scaffolds were cut into approximately 0.5 × 0.5 cm pieces and attached to the bottom of the 24 multi-well plate (Nunc) using lip balm. The cells were seeded at a density of 100 000 cells per scaffold and left for 1 h in the incubator before adding the complete cell culture medium (DMEM/F12 complete medium containing the growth factors B27 supplement, bFGF and EGF), to allow cell attachment to the surface of the scaffolds.

In the case of PLA and PLA/GnP coverslips, the number of PKH^POS^ seeded cells was 16 000 per coated coverslip. Cells were left for 1 h in the incubator to allow cell attachment and complete cell culture medium added. Experiments were performed in duplicates and *n* = 3 independent experiments. The cells were kept in culture for 1 day, 5 days, 7 days and 14 days depending of the required analysis procedure.

### Scanning electron microscopy (SEM) imaging

PKH^pos^ cells plated on the various substrates, were fixed with Karnovsky's fixative (2% paraformaldehyde; 2.5% glutaraldehyde in 0.1 M phosphate buffer pH 7.4 for 2 h, and washed three times with Na-cacodylate buffer 0,1 M. After fixation, a post-fixative step with 1% osmium tetroxide in 0.1 M of cacodylate buffer 2 h at room temperature was applied, followed by a dehydration protocol with 50%, 75%, 95% ethanol to water ratio (in volume) and then absolute ethanol. Finally, samples were coated with 10 nm Au and observed at SEM (JEOL JSM-6490LA).

### Electrophysiological recordings on PKH^pos^ CSCs and differentiated cells

Resting membrane potentials were recorded in the whole-cell configuration of the patch-clamp technique at room temperature. The choice of the cells to record was based on PKH fluorescent signal. External recording solution contained (in mM): 145 NaCl, 2 KCl, 2 CaCl_2_, 2 MgCl_2_, 10 glucose, and 10 HEPES, pH 7.4. Patch pipettes, pulled from borosilicate glass capillaries (Warner Instruments, LLC, Hamden, USA) had a 4 to 5 MΩ resistance when filled with intracellular solution. In all experiments, the intracellular solution contained (in mM): 10 KGluconate, 125 KCl, 1 EGTA, 10 HEPES, 5 sucrose, 4 MgATP (300 mOsm and pH 7.2 with KOH). Membrane potentials were recorded using Clampex 10.0 software (Molecular Devices, Sunnyvale, CA). The stability of the patch was checked by monitoring the input resistance during the experiments to exclude cells exhibiting more than 15% changes from the analysis.

### Immunostaining and confocal analysis

U87 CSCs PKH^POS^ seeded on scaffolds, were fixed with paraformaldehyde solution 4% in PBS buffer 1× pH = 7.4 for 20 min. After this incubation time, cells were washed three times with PBS 1× buffer. The cells were incubated in a solution with 10% bovine serum albumin (Sigma A9418-10G), 0.25% Triton X-100 (Sigma X100-100ML) (percentage in volume) and PBS 1× buffer pH = 7.4 for 1 h, at room temperature. All the primary and secondary antibodies were diluted in 1% of bovine serum albumin, 0.25% Triton X-100 in PBS 1× pH = 7.4 buffer, Sigma solution (volume dilution). Anti-glial fibrillary acidic protein (GFAP) rabbit antibody (1 : 500 diluted; Sigma G9269-25UL) or anti-nestin mouse antibody (1 : 100 diluted; Invitrogen, MA1-110) or anti-S100B rabbit antibody (1 : 200 diluted; Sigma HPA015768) were used like primary antibodies, incubated overnight at 4 °C. Cells were then washed three times with PBS 1× pH = 7.4, then incubated with the secondary anti-rabbit antibody Texas red conjugated (Invitrogen, T-2767; 1 : 1000 diluted) or anti-mouse antibody Texas red conjugated (Invitrogen T-6390; 1 : 1000 diluted) or anti-rabbit antibody Alexa Fluor 405 conjugated (Invitrogen A-31556, 1 : 200 diluted) (volume dilution) for 1 h 15 min at room temperature. The cells were finally washed three times with PBS 1× pH = 7.4. Subsequently, cells were incubated with a DAPI solution ((Roche-10236276001; 1 : 500) diluted in a solution of 0.25% Triton X-100 and PBS 1× pH = 7.4 buffer and incubated for 30 min at room temperature. After the incubation time, cells were washed three times with PBS 1× pH = 7.4 buffer, scaffolds were dried at room temperature and mounted with Prolong Antifade reagent (Life Technologies) and analyzed by a confocal fluorescence microscope (A1+ confocal microscope system, Nikon). Image acquisition and processing were conducted by Nikon NIS Elements software, at an excitation/emission wavelength of 358/461 nm for DAPI (blue), 401/421 nm for Alexa Fluor 405 (blue), and 586/603 nm for Texas red dye (red).

### Numerical calculations

COMSOL Multiphysics, a finite element method-based software, was employed for the numerical electrostatic simulations. A spherical domain of varying diameter representing the GnP was placed in contact with a 10 nm layer (*z*-direction) representing the membrane. A PA66 layer of 5 nm has been considered around the GnP. The baseline electric potential across the membrane has been set to −10 mV (−10 mV on the inside and 0 mV on the outside, facing the GnP). An additional electrical potential (varying as described in the main text) has been subsequently applied to the surface of the GnP to mimic the presence of charge. The total potential has been calculated as the sum of the membrane and GnP potential. Both the PA66 substrate below the GnP and the inner cell cytoplasm have been truncated by means of infinite elements along the *z*-direction. The lateral sizes (*xy*-plane) have been extended for 100 μm to avoid any spurious boundary effect. Zero charge boundary conditions have been utilized. The GnP has been meshed with tetrahedral elements with maximum size equal to 1/10 of the GnP diameter. The membrane has been meshed vertically (*z*-direction) with 1 nm thick elements. Tetrahedral elements have been utilized to complete the rest of the mesh. The different domains have been characterized through their electric permittivity, *ε*. In particular: *ε*_GnP_ = 10, *ε*_membrane_ = 2.5, *ε*_cytoplasm_ = 80, *ε*_nylon_ = 3.6.

## Author contributions

EPL, RC and RPZ conceived the idea. ELP and AD performed electrospinning of PA66. ELP performed electrospinning of PLA, Raman, XR SEM. LC did the mechanical testing. TC did TEM. MS did AFM and Kelvin probe. MC designed and built the digital electroscope. RPZ and AlA performed the numerical simulations. TFC and AEM prepared cell cultures for SEM, electrophysiology and immunostaining analysis, performed PKH staining, FACS, immunostaining and confocal microscopy. MR, EMP and AB did the electrophysiology. ELP and TFC wrote the first draft of the manuscript. ELP, TP and AtA contributed in the writing and supervised the project.

## Conflicts of interest

There are no conflicts to declare.

## Supplementary Material

NH-008-D2NH00453D-s001

## References

[cit1] Blackiston D. J., McLaughlin K. A., Levin M. (2009). Bioelectric controls of cell proliferation: Ion channels, membrane voltage and the cell cycle. Cell Cycle.

[cit2] Sundelacruz S., Levin M., Kaplan D. L. (2008). Membrane Potential Controls Adipogenic and Osteogenic Differentiation of Mesenchymal Stem Cells. PLoS One.

[cit3] Sundelacruz S., Levin M., Kaplan D. L. (2009). Role of membrane potential in the regulation of cell proliferation and differentiation. Stem Cell Rev. Rep..

[cit4] van Vliet P. (2010). *et al.*, Hyperpolarization induces differentiation in human cardiomyocyte progenitor cells. Stem Cell Rev. Rep..

[cit5] Bhavsar M. B. (2020). *et al.*, Role of Bioelectricity During Cell Proliferation in Different Cell Types. Front. Bioeng. Biotechnol..

[cit6] Levin M. (2012). Molecular bioelectricity in developmental biology: new tools and recent discoveries: control of cell behavior and pattern formation by transmembrane potential gradients. BioEssays.

[cit7] Levin M. (2013). Reprogramming cells and tissue patterning *via* bioelectrical pathways: molecular mechanisms and biomedical opportunities. Wiley Interdiscip. Rev.: Syst. Biol. Med..

[cit8] Yang M., Brackenbury W. J. (2013). Membrane potential and cancer progression. Front. Physiol..

[cit9] Mirsadeghi S. (2017). *et al.*, Development of membrane ion channels during neural differentiation from human embryonic stem cells. Biochem. Biophys. Res. Commun..

[cit10] Konig S. (2006). *et al.*, The calcineurin pathway links hyperpolarization (Kir2.1)-induced Ca^2+^ signals to human myoblast differentiation and fusion. Development.

[cit11] Konig S. (2004). *et al.*, Membrane hyperpolarization triggers myogenin and myocyte enhancer factor-2 expression during human myoblast differentiation. J. Biol. Chem..

[cit12] Dante S. (2017). *et al.*, Selective Targeting of Neurons with Inorganic Nanoparticles: Revealing the Crucial Role of Nanoparticle Surface Charge. ACS Nano.

[cit13] Distasi C. (2018). *et al.*, SiO2 nanoparticles modulate the electrical activity of neuroendocrine cells without exerting genomic effects. Sci. Rep..

[cit14] Jayaram D. T. (2017). *et al.*, Controlling the Resting Membrane Potential of Cells with Conducting Polymer Microwires. Small.

[cit15] Moschetta M. (2021). *et al.*, Graphene Nanoplatelets Render Poly(3-Hydroxybutyrate) a Suitable Scaffold to Promote Neuronal Network Development. Front. Neurosci..

[cit16] Yamada M. (2007). *et al.*, Electrical stimulation modulates fate determination of differentiating embryonic stem cells. Stem Cells.

[cit17] Rahmani A. (2019). *et al.*, Conductive electrospun scaffolds with electrical stimulation for neural differentiation of conjunctiva mesenchymal stem cells. Artif. Organs.

[cit18] Crowder S. W. (2016). *et al.*, Material Cues as Potent Regulators of Epigenetics and Stem Cell Function. Cell Stem Cell.

[cit19] Huang Y. J. (2012). *et al.*, Carbon nanotube rope with electrical stimulation promotes the differentiation and maturity of neural stem cells. Small.

[cit20] Ostrakhovitch E. A. (2012). *et al.*, Directed differentiation of embryonic P19 cells and neural stem cells into neural lineage on conducting PEDOT-PEG and ITO glass substrates. Arch. Biochem. Biophys..

[cit21] Wang Y. (2012). *et al.*, Fluorinated Graphene for Promoting Neuro-Induction of Stem Cells. Adv. Mater..

[cit22] Guo R. (2016). *et al.*, Accelerating bioelectric functional development of neural stem cells by graphene coupling: Implications for neural interfacing with conductive materials. Biomaterials.

[cit23] Scapin G. (2015). *et al.*, Enhanced neuronal cell differentiation combining biomimetic peptides and a carbon nanotube-polymer scaffold. Nanomedicine.

[cit24] Scapin G. (2016). *et al.*, Neuronal commitment of human circulating multipotent cells by carbon nanotube-polymer scaffolds and biomimetic peptides. Nanomedicine.

[cit25] Li N. (2013). *et al.*, Three-dimensional graphene foam as
a biocompatible and conductive scaffold for neural stem cells. Sci. Rep..

[cit26] Solanki A. (2013). *et al.*, Axonal Alignment and Enhanced Neuronal Differentiation of Neural Stem Cells on Graphene-Nanoparticle Hybrid Structures. Adv. Mater..

[cit27] Kim J. (2015). *et al.*, Monolayer Graphene-Directed Growth and Neuronal Differentiation of Mesenchymal Stem Cells. J. Biomed. Nanotechnol..

[cit28] Nayak T. R. (2011). *et al.*, Graphene for controlled and accelerated osteogenic differentiation of human mesenchymal stem cells. ACS Nano.

[cit29] Wang Y. (2012). *et al.*, Fluorinated graphene for promoting neuro-induction of stem cells. Adv. Mater..

[cit30] Kostarelos K., Novoselov K. S. (2014). Materials science. Exploring the interface of graphene and biology. Science.

[cit31] Bramini M. (2018). *et al.*, Interfacing Graphene-Based Materials With Neural Cells. Front. Syst. Neurosci..

[cit32] Schiffer D. (2018). *et al.*, Glioblastoma: Microenvironment and Niche Concept. Cancers.

[cit33] Maier-Hauff K. (2011). *et al.*, Efficacy and safety of intratumoral thermotherapy using magnetic iron-oxide nanoparticles combined with external beam radiotherapy on patients with recurrent glioblastoma multiforme. J. Neurooncol..

[cit34] Persano S. (2021). *et al.*, Elucidating the Innate Immunological Effects of Mild Magnetic Hyperthermia on U87 Human Glioblastoma Cells: An In Vitro Study. Pharmaceutics.

[cit35] Gupta R., Sharma D. (2019). Evolution of Magnetic Hyperthermia for Glioblastoma Multiforme Therapy. ACS Chemical. Neuroscience.

[cit36] Gavilán H. (2021). *et al.*, Magnetic nanoparticles and clusters for magnetic hyperthermia: optimizing their heat performance and developing combinatorial therapies to tackle cancer. Chem. Soc. Rev..

[cit37] Lathia J. D. (2015). *et al.*, Cancer stem cells in glioblastoma. Genes Dev..

[cit38] Jian Z. (2017). *et al.*, Cancer Stem Cells in Squamous Cell Carcinoma. J. Invest. Dermatol..

[cit39] Fiorillo M. (2015). *et al.*, Graphene oxide selectively targets cancer stem cells, across multiple tumor types: implications for non-toxic cancer treatment, *via* “differentiation-based nano-therapy”. Oncotarget.

[cit40] Gurunathan S., Kim J. H. (2017). Graphene Oxide-Silver Nanoparticles Nanocomposite Stimulates Differentiation in Human Neuroblastoma Cancer Cells (SH-SY5Y). Int. J. Mol. Sci..

[cit41] Verre A. F. (2018). *et al.*, Improving the glial differentiation of human Schwann-like adipose-derived stem cells with graphene oxide substrates. Interface Focus.

[cit42] Jaworski S. (2019). *et al.*, Degradation of Mitochondria and Oxidative Stress as the Main Mechanism of Toxicity of Pristine Graphene on U87 Glioblastoma Cells and Tumors and HS-5 Cells. Int. J. Mol. Sci..

[cit43] Wierzbicki M. (2017). *et al.*, Diamond, graphite, and graphene oxide nanoparticles decrease migration and invasiveness in glioblastoma cell lines by impairing extracellular adhesion. Int. J. Nanomed..

[cit44] Szmidt M. (2019). *et al.*, Graphene oxide down-regulates genes of the oxidative phosphorylation complexes in a glioblastoma. BMC Mol. Biol..

[cit45] Szczepaniak J. (2018). *et al.*, Effects of Reduced Graphene Oxides on Apoptosis and Cell Cycle of Glioblastoma Multiforme. Int. J. Mol. Sci..

[cit46] Patro N., Naik A., Patro I. K. (2015). Differential temporal expression of S100β in developing rat brain. Front. Cell. Neurosci..

[cit47] Yoo S. J. (2011). *et al.*, Simple and novel three dimensional neuronal cell culture using a micro mesh scaffold. Exp. Neurobiol..

[cit48] Papadopoulou E. L. (2016). *et al.*, Nylon 6,6/graphene nanoplatelet composite films obtained from a new solvent. RSC Adv..

[cit49] Li Z. and Wang C., One-Dimensional Nanostructures: Electrospinning Technique and Unique Nanofibers, Briefs in Materials, Springer, 2013

[cit50] Bramini M. (2016). *et al.*, Graphene Oxide Nanosheets Disrupt Lipid Composition, Ca(2+) Homeostasis, and Synaptic Transmission in Primary Cortical Neurons. ACS Nano.

[cit51] Schoen I., Fromherz P. (2007). The mechanism of extracellular stimulation of nerve cells on an electrolyte-oxide-semiconductor capacitor. Biophys. J..

[cit52] Diaz A. F., Felix-Navarro R. M. (2004). A semi-quantitative tribo-electric series for polymeric materials: the influence of chemical structure and properties. J. Electrost..

[cit53] Pinto T. M., Wedemann R. S., Cortez C. M. (2014). Modeling the electric potential across neuronal membranes: the effect of fixed charges on spinal ganglion neurons and neuroblastoma cells. PLoS One.

[cit54] Gimsa J. (1996). *et al.*, Dielectric spectroscopy of single human erythrocytes at physiological ionic strength: dispersion of the cytoplasm. Biophys. J..

[cit55] Cataldi P. (2016). *et al.*, Effect of graphene nano-platelet morphology on the elastic modulus of soft and hard biopolymers. Carbon.

[cit56] Fernandes S. (2021). *et al.*, Magnetic Nanoparticle-Based Hyperthermia Mediates Drug Delivery and Impairs the Tumorigenic Capacity of Quiescent Colorectal Cancer Stem Cells. ACS Appl. Mater. Interfaces.

[cit57] Pece S. (2010). *et al.*, Biological and Molecular Heterogeneity of Breast Cancers Correlates with Their Cancer Stem Cell Content. Cell.

[cit58] Vicentini N. (2018). *et al.*, Effect of different functionalized carbon nanostructures as fillers on the physical properties of biocompatible poly(l-lactic acid) composites. Mater. Chem. Phys..

[cit59] Scapin G. (2016). *et al.*, Neuronal commitment of human circulating multipotent cells by carbon nanotube-polymer scaffolds and biomimetic peptides. Nanomedicine.

[cit60] Pan R. (2018). *et al.*, Fully biodegradable triboelectric nanogenerators based on electrospun polylactic acid and nanostructured gelatin films. Nano Energy.

[cit61] Soto-Cerrato V. (2015). *et al.*, Facilitated Anion Transport Induces Hyperpolarization of the Cell Membrane That Triggers Differentiation and Cell Death in Cancer Stem Cells. J. Am. Chem. Soc..

[cit62] Bautista W. (2017). *et al.*, Cancer Stem Cells are Depolarized Relative to Normal Stem Cells Derived from Human Livers. Ann. Hepatol..

[cit63] Collins G. (2012). *et al.*, Charge generation, charge transport, and residual charge in the electrospinning of polymers: A review of issues and complications. J. Appl. Phys..

[cit64] Diao W. (2019). *et al.*, Behaviors of Glioblastoma Cells in in Vitro Microenvironments. Sci. Rep..

[cit65] Neradil J., Veselska R. (2015). Nestin as a marker of cancer stem cells. Cancer Sci..

[cit66] Generazio E. R. (2017). Electric potential and electric field imaging. AIP Conf. Proc..

