# Supplementary material for: Electrostatic polarization fields trigger glioblastoma stem cell differentiation
Source: Nanoscale Horiz. 2022 Nov 17;8(1):95–107. doi: 10.1039/d2nh00453d (PMC9765404; doi:10.1039/d2nh00453d)
Supplement: NH-008-D2NH00453D-s001 [file NH-008-D2NH00453D-s001.pdf]

Electronic Supplementary Material (ESI) for Nanoscale Horizons.
This journal is © The Royal Society of Chemistry 2022

Supplementary Information

Electrostatic polarization fields trigger glioblastoma stem cell differentiation

Tamara Fernandez Cabada[1], Massimo Ruben[1,i], Amira El Merhie[1,ii], Remo Proietti Zaccaria[1,*], Alessandro Alabastri[2], Enrica Maria Petrini[1,*], Andrea Barberis[1], Marco Salerno[1], Marco Crepaldi[3], Alexander Davis[1], Luca Ceseracciu[1], Tiziano Catelani[1,iii], Athanassia Athanassiou[1], Teresa Pellegrino[1,*] Roberto Cingolani[1,iv] and Evie L. Papadopoulou[1,*]

[1] Istituto Italiano di Tecnologia, via Morego 30, 16163 Genova, Italy

[2] Department of Electrical and Computer Engineering, Rice University, 6100 Main Street, Houston, TX, 77005 USA

[3] Istituto Italiano di Tecnologia, via Melen 83, 16152 Genova, Italy

[i] *Current address:* University of Bordeaux, CNRS, Interdisciplinary Institute for Neuroscience, IINS, UMR 5297, 33000 Bordeaux, France

[ii] Current address: Mines Saint-Etienne, Centre CMP, Department of Bioelectronics, Gardanne, France

[iii] *Current Address:* Electron Microscopy and Micro-Analysis Facility (MEMA), University of Florence, via G. Capponi, 3r - 50121 Firenze, Italy

[iv] *Current Address:* Leonardo Company, Piazza Monte Grappa 4, 00195 Rome, Italy

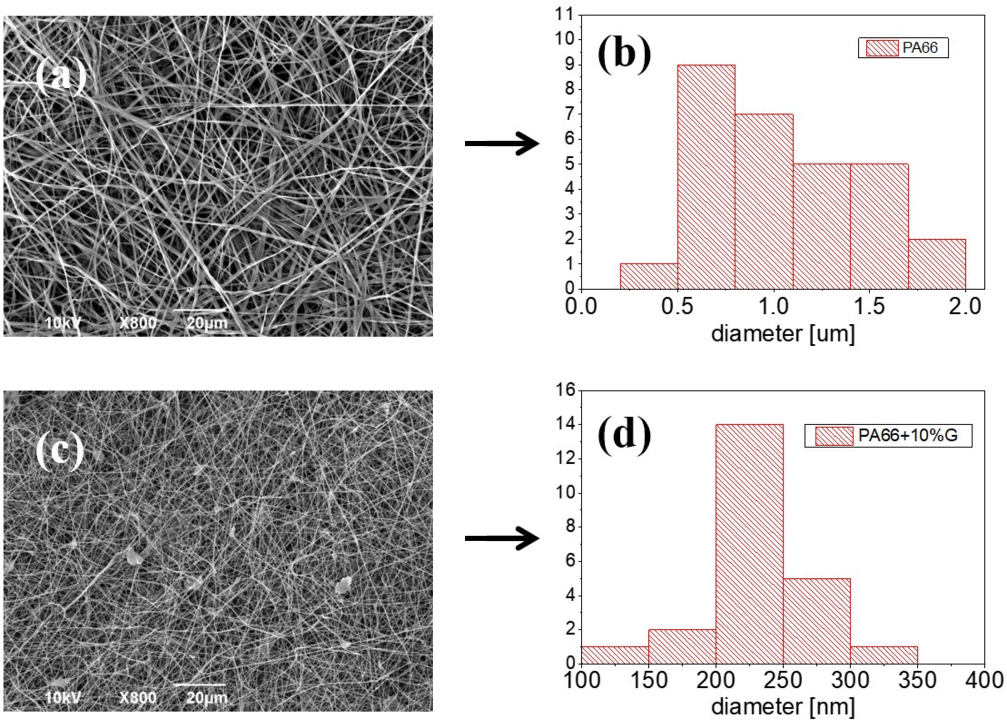

Figure S1: (a) SEM of PA66 fibres, (b) diameter distribution of fibres presented in (a), (c) SEM of PA66/G fibres, (d) diameter distribution of fibres presented in (c).

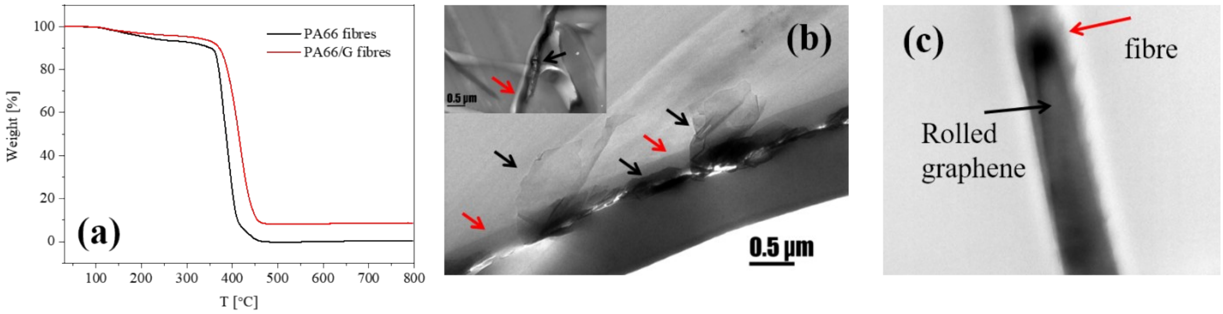

Figure S2: (a) The thermal degradation of the composites measured by Thermogravimetric Analysis (TGA). The degradation of PA66 takes place between 350 °C and 500 °C, and practically nothing remains at 600 C (<0.01%). In the case of PA66/GnP the weight loss is gradual and the remaining material at 600 °C is about 8.5%, indicating the percentage of GnPs present in the composite (nominal value 10%); (b) and (c) TEM images of single fibres showing the GnP either (c) rolled inside the PA66 fibre, or (d) piercing out of the fibre (red arrows indicate the fibre, black arrows indicate the GnP).

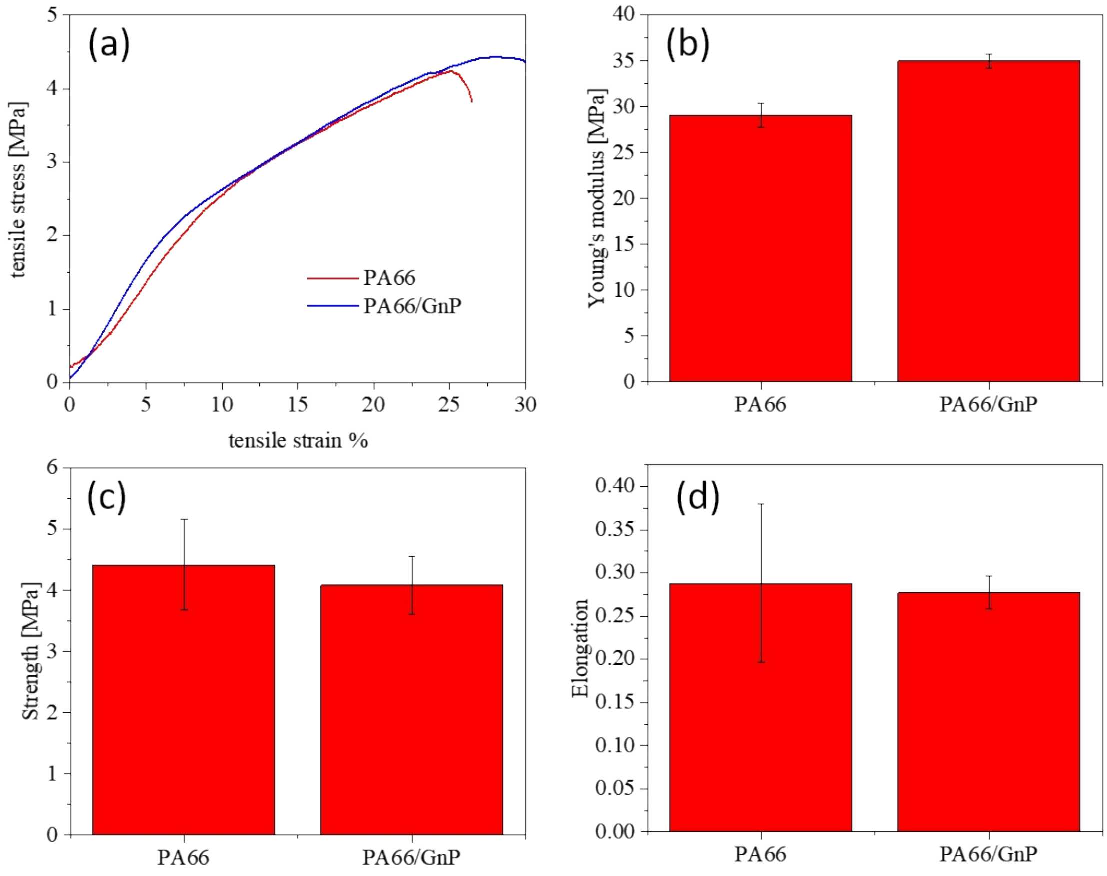

Figure S3: (a) Stress-strain curves for PA66 and PA66/GnP fibre mats, (b) the Young's modulus, (c) the strength and (d) the elongation of the PA66 and the PA66/GnP fibre mats. While the Young's modulus is seen to slightly increase with the inclusion of GnP in the PA66 fibre mats, the strength and the elongation remain similar.

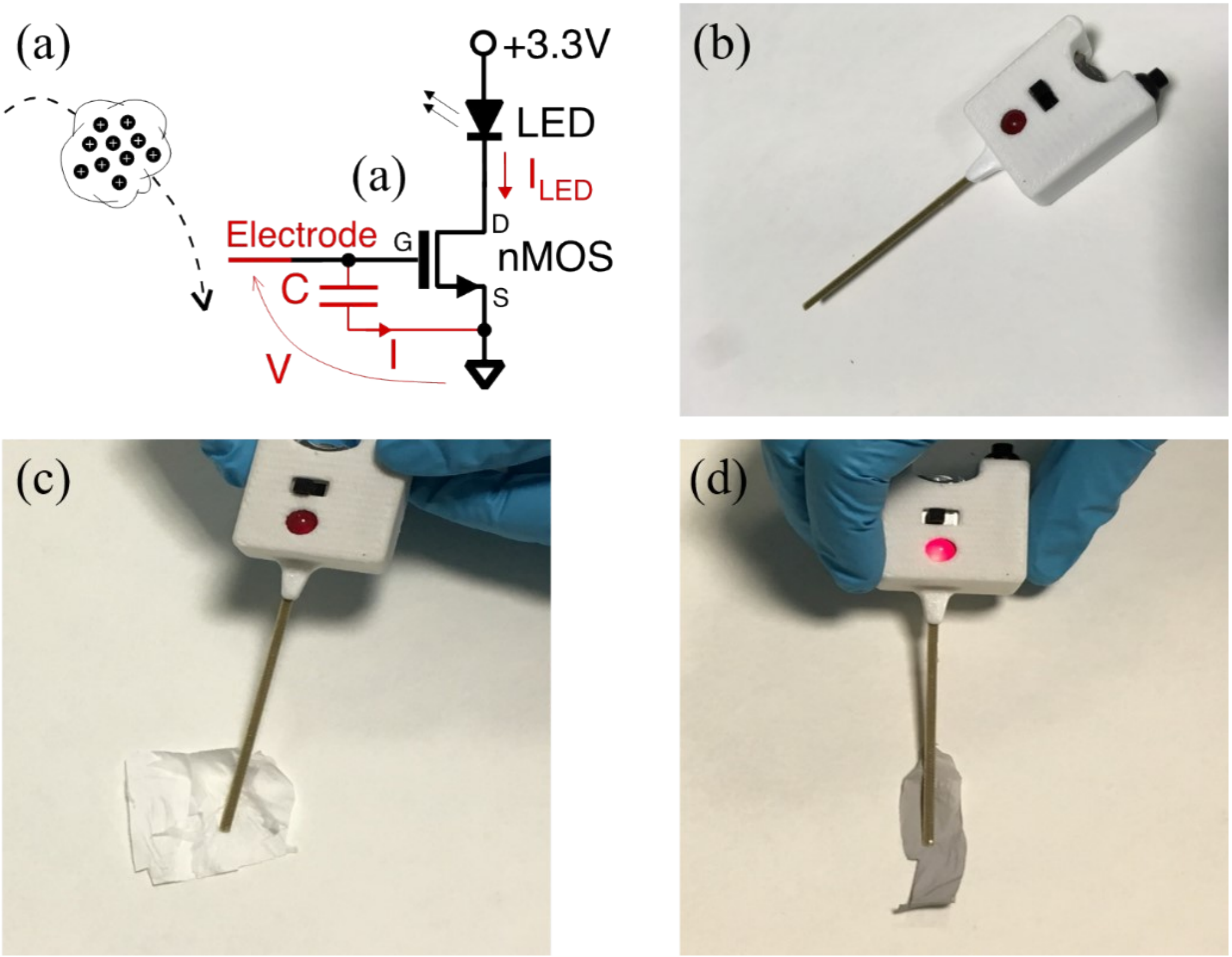

Figure S4: (a) Basic schematic of the digital electroscope, (b) detector prototype photo, powered by two CR1220 batteries. Voltage regulator and internal schematic not shown for the sake of brevity. The circuit is packaged in a Polyacetic Acid (PLA) case, (c) The detector approaching PA66 fibres where there is a slight change in the light intensity, indicating positive charges close to zero, and (d) the detector approaching PA66/GnP fibres and the light intensity increases indicating positive charges on the surface.

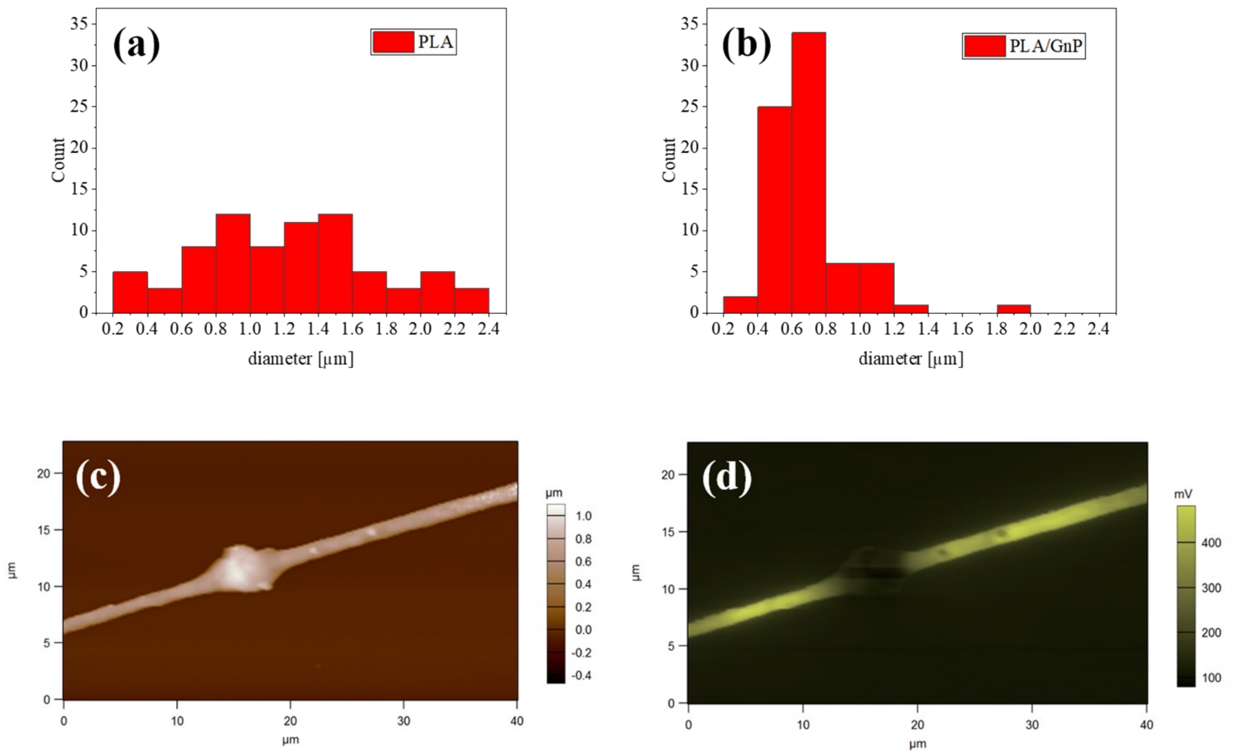

Figure S5: Diameter distribution for (a) PLA and (b) PLA/GnP electrospun fibres.(c) Representative AFM topography image of a PLA fibre, with (d) its corresponding surface electrical potential map, as measured by Kelvin probe AFM. The colour bars on the right show the range of surface potential values within each image

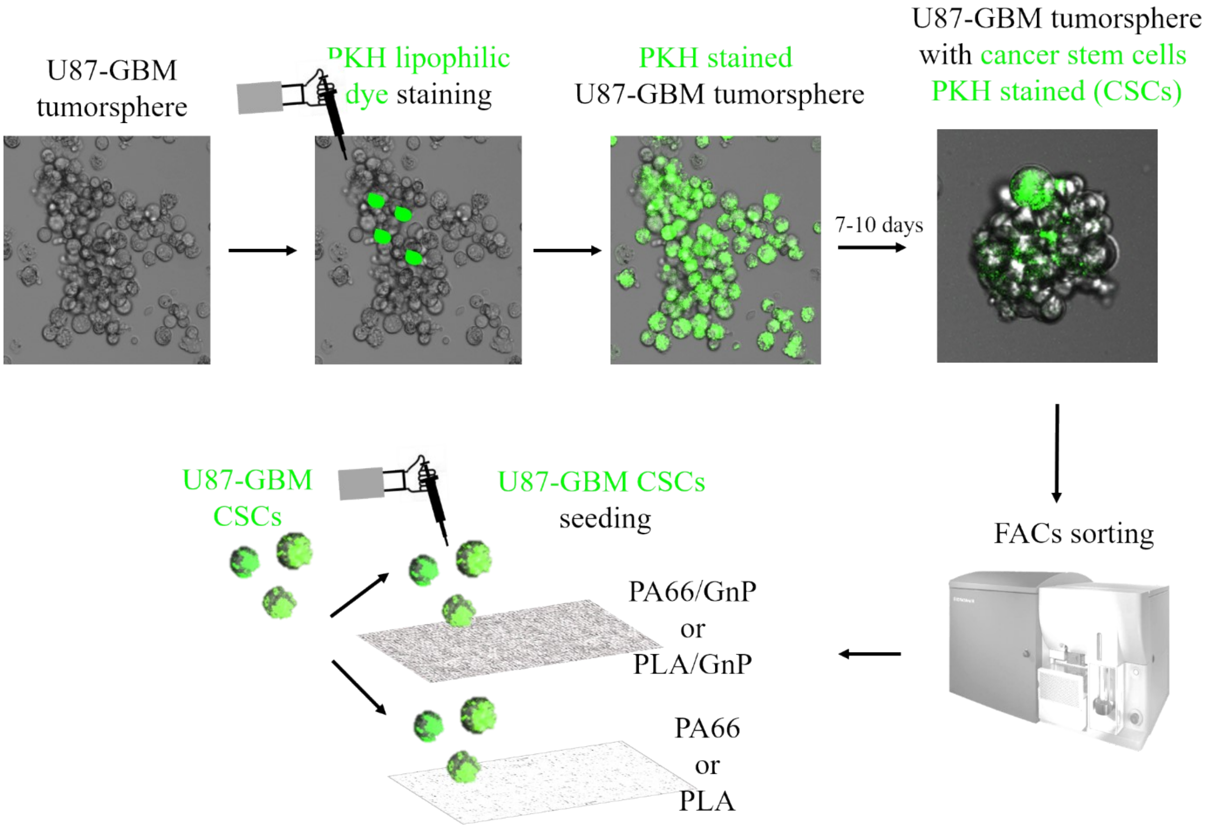

Figure S6: Sketch of the experimental procedure. PKH lipophilic dye staining procedure of the U87 GBM tumorspheres. After DIV7-10, only quiescent cells (U87 CSCs) keep the PKH dye. Based on that principle, U87 CSCs are sorted by FACs.

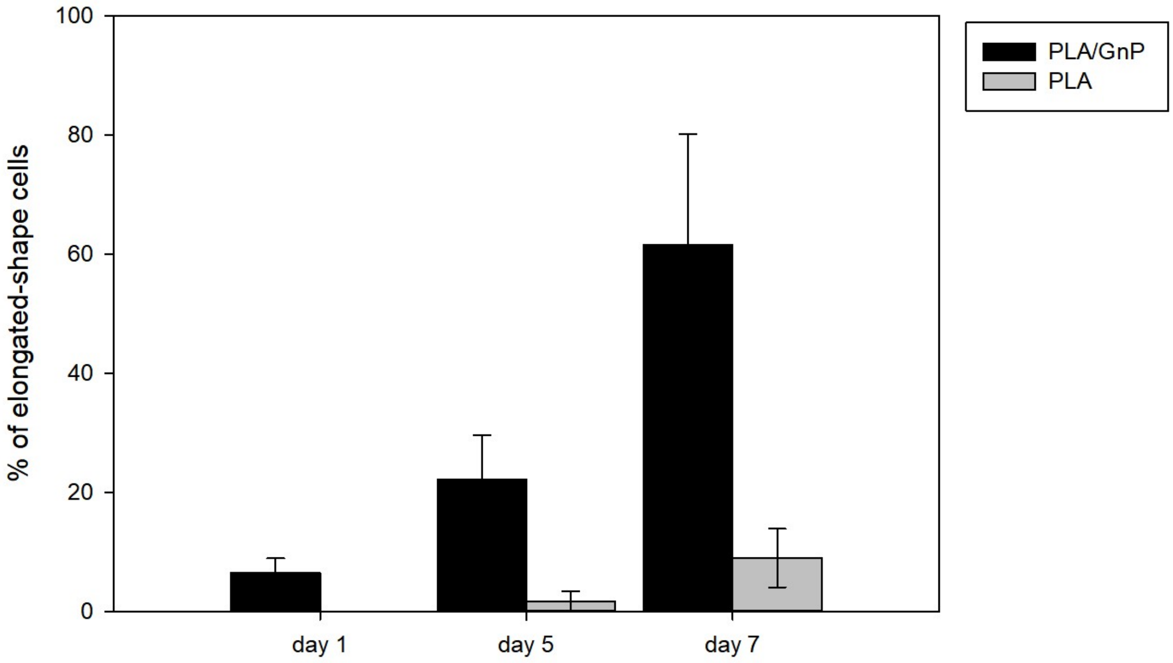

Figure S7: Percentage of elongated cells with respect to the total number of cells (elongated plus rounded cells). The values correspond to the mean ± SD of n = 100 cells per each experimental condition, in n=3 independent experiments. Statistical analysis was done using One way ANOVA and Dunn´s multiple comparison test, with ***p<0,001 in each time point. On DIV1 the PLA bar is missing because the amount of elongated cells is null.

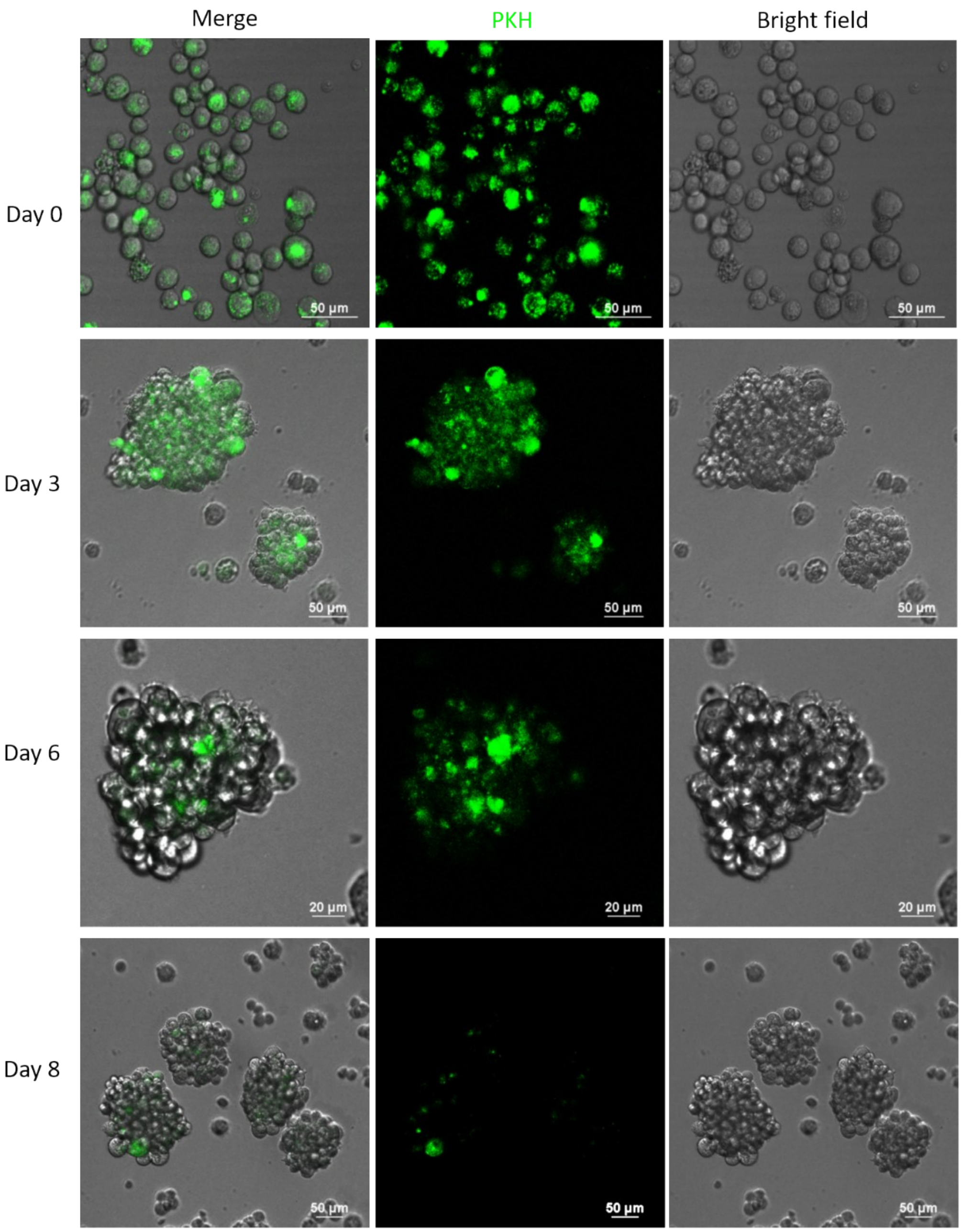

Figure S8: Spheroid formation.
